# Bedbugs as Vectors for Drug-Resistant Bacteria

**DOI:** 10.3201/eid1706.101978

**Published:** 2011-06

**Authors:** Christopher F. Lowe, Marc G. Romney

**Affiliations:** Author affiliations: University of Toronto, Toronto, Ontario, Canada (C.F. Lowe);; University of British Columbia, Vancouver, British Columbia, Canada (M.G. Romney);; St. Paul’s Hospital, Providence Health Care, Vancouver (M.G. Romney)

**Keywords:** bedbug, Cimex lectularius, MRSA, VRE, methicillin-resistant Staphylococcus aureus, staphylococci, vancomycin-resistant Enterococcus faecium, antimicrobial resistance, bacteria, letter

**To the Editor:** Over the past 10 years in North America and western Europe, a resurgence of bedbugs (*Cimex lectularius*) has occurred (*1*). Although the basis for this resurgence is unclear, large bedbug infestations have been attributed to increased worldwide travel, altered insecticide management, and increased resistance to pesticides (*2*). Marginalized populations in large urban centers appear to be disproportionately affected; ≈30% of shelters in Toronto, Ontario, Canada, report bedbugs (*1*). We report recovery of methicillin-resistant *Staphylococcus aureus* (MRSA) and vancomycin-resistant *Enterococcus faecium* (VRE) from bedbugs in Vancouver, British Columbia.

Three patients, all residents of Vancouver’s Downtown Eastside—an impoverished community in Vancouver with high rates of homelessness, poverty, HIV/AIDS, and injection drug use (*3*)—were hospitalized and found to be infested with bedbugs. Hypothesizing that these parasites may be vectors for the transmission of antimicrobial drug–resistant pathogens, we collected 5 bedbugs and tested them for drug-resistant organisms. The bedbugs were homogenized and streaked onto standard microbiological media, including 5% sheep blood agar. Bacterial colonies were identified by using conventional and automated microbiological methods, and susceptibility testing was performed in accordance with Clinical Laboratory Standards Institute guidelines (*4*). For 2 patients, VRE was isolated from 1 bedbug each. These bacterial isolates were also resistant to ampicillin, teicoplanin, and aminoglycosides but susceptible to linezolid, quinupristin/dalfopristin, and tetracycline. For 1 other patient, MRSA was isolated from 3 bedbugs. All MRSA isolates had susceptibility patterns consistent with pulsed-field gel electrophoresis type USA300 (susceptible to vancomycin, clindamycin, trimethoprim/sulfamethoxasole, tetracycline, and rifampin; resistant to erythromycin).

Despite investigations of transmissibility of numerous infectious agents, including transmissible blood-borne pathogens such as HIV and hepatitis B and C viruses, to our knowledge, no conclusive evidence has demonstrated disease transmission by bedbugs (*5**,**6*). Clinically, bedbug bites have been associated with cutaneous manifestations, most commonly pruritic wheals with a central hemorrhagic punctum (*6*). Excoriation at the site of a bite can cause further skin abrasion, thereby providing an entry point for colonizing bacteria, which can potentially result in folliculitic or cellulitic superinfections (*5*). *S. aureus*, which is commonly found on the skin and can cause cellulitis, has been reported to colonize the salivary glands of bedbugs for as long as 15 days (*7*). However, although transient and persistent forms of colonization may play a role in disease transmission, we did not differentiate these forms because the clinical bedbug specimens were processed at the time of receipt in the laboratory.

Similar to other cities worldwide, Vancouver has seen an alarming increase in bedbugs, particularly in the Downtown Eastside, where 31% of residents have reported bedbug infestation (*8*). Enterococci commensally occupy the gastrointestinal tract. The recovery of VRE from bedbugs could result from the relatively unhygienic living conditions encountered in the Downtown Eastside and the high level of interconnectedness between St. Paul’s Hospital (where a large pool of patients with VRE colonization/infection reside) and the surrounding community.

MRSA is also a substantial problem in the Downtown Eastside; it has been cultured from 54.8% of skin and soft tissue infections of patients seen at the emergency department of St. Paul’s Hospital (*9*). More recently, among wound infections in injection drug users in this community, 43% were colonized or infected with MRSA consistent with USA300, the predominant community-associated MRSA strain in Vancouver (*10*). The phenotype of the MRSA recovered from the bedbugs was consistent with community-associated MRSA and identical to that found on antibiograms from patients with MRSA infection who reside in this community. Given the high prevalence of MRSA (particularly USA300) in hotels and rooming houses in Vancouver’s Downtown Eastside, bedbugs may become colonized with community-associated MRSA. Consequently, these insects may act as a hidden environmental reservoir for MRSA and may promote the spread of MRSA in impoverished and overcrowded communities.

Bedbugs carrying MRSA and/or VRE may have the potential to act as vectors for transmission. Further studies are needed to characterize the association between *S. aureus* and bedbugs. Bedbug carriage of MRSA, and the portal of entry provided through feeding, suggests a plausible potential mechanism for passive transmission of bacteria during a blood meal. Because of the insect’s ability to compromise the skin integrity of its host, and the propensity for *S. aureus* to invade damaged skin, bedbugs may serve to amplify MRSA infections in impoverished urban communities.
